# Detailed phylogenetic analysis tracks transmission of distinct SARS-COV-2 variants from China and Europe to West Africa

**DOI:** 10.1038/s41598-021-00267-w

**Published:** 2021-10-26

**Authors:** Wasco Wruck, James Adjaye

**Affiliations:** Institute for Stem Cell Research and Regenerative Medicine, Medical Faculty, Heinrich Heine University, 40225 Düsseldorf, Germany

**Keywords:** Phylogeny, Viral infection

## Abstract

SARS-CoV-2, the virus causing the COVID-19 pandemic emerged in December 2019 in China and raised fears it could overwhelm healthcare systems worldwide. Mutations of the virus are monitored by the GISAID database from which we downloaded sequences from four West African countries Ghana, Gambia, Senegal and Nigeria from February 2020 to April 2020. We subjected the sequences to phylogenetic analysis employing the nextstrain pipeline. We found country-specific patterns of viral variants and supplemented that with data on novel variants from June 2021. Until April 2020, variants carrying the crucial Europe-associated D614G amino acid change were predominantly found in Senegal and Gambia, and combinations of late variants with and early variants without D614G in Ghana and Nigeria. In June 2021 all variants carried the D614G amino acid substitution. Senegal and Gambia exhibited again variants transmitted from Europe (alpha or delta), Ghana a combination of several variants and in Nigeria the original Eta variant. Detailed analysis of distinct samples revealed that some might have circulated latently and some reflect migration routes. The distinct patterns of variants within the West African countries point at their global transmission via air traffic predominantly from Europe and only limited transmission between the West African countries.

## Introduction

The COVID-19 pandemic resulting from the SARS-CoV-2 coronavirus infection which emerged in December 2019 in Wuhan, China, spread all over the world and after a delay of a few months also appeared on the African continent. Early in June 2020, all African countries registered human infections with SARS-CoV-2. Starting with the first sequenced human sample of SARS-CoV-2, several mutations of the virus sequence arose which could be grouped into clades allowing associations with regional prevalence. In this study, we focused on samples from West Africa which was the region from which the first African SARS-CoV-2 sequences became available. We aimed at analysing phylogenetic characteristics possibly giving hints on the distribution between countries and eventually even about putative severity changes between specific clades. We use the nomenclature of clades suggested by the GISAID initiativewhich has now been adopted in several publications. Previous studies reported the potential impact of the D614G amino acid substitution which is a result of the A23403G single nucleotide polymorphism (SNP)^1^ and associated with the branch of the phylogenetic tree referred to as clade G.

Brufsky hypothesized that the higher number of deaths on the East coast of the United States compared to the West coast could be due to the higher prevalence of the D614G amino acid substitution on the East coast^2^. The D614G amino acid substitution has been suggested to affect the adherence of the virus to the cell membrane and consequently results in higher virulence. Supportive evidence was reported in mice^3,4^. Korber et al. hypothesized that the D614 amino acid on the surface of the subunit S1 of the spike protein of the virus might have a hydrogen bond to the T859 amino acid in the subunit S2 residing on the membrane^1^. Furthermore, they showed that clade G rapidly starts to replace other clades associated with the D614 amino acid in each country entered^1^. In mid-March 2020, the G clade was found almost exclusively in Europe^5^ but soon after spread all over the world. Korber et al. see its origin from China or Europe^1^. In China, four early samples carried the D614G amino acid substitution. One sample from January 24th 2020 had only the A23403G (D614G) but not the C3037T and C14408T mutations which usually associate with A23403T in clade G. Three samples with the D614G were related to the first German sample. In Europe the first German sample from January 28th carried the A-to-G mutation at nucleotide position 23403 (D614G) mutation and the C-to-T mutation at position 3037, but not the mutation at position 14408. The first sample carrying all of the above mentioned mutations plus the C241T in the Untranslated Region (UTR) was identified in Italy on February 20th, 2020^1^. Another interesting feature of the G clades is that the associated C14408T mutation adjacent to the RNA dependent RNA polymerase (RdRp) putatively increases the mutation rate as Pachetti et al*.* report^5^.

Detailed analyses of virus evolution have been performed for some countries, e.g. France^6^, New York^7^ and India^8^. For France it could be deduced by the distinction between clade G and the earlier phylogenetic branches that the first SARS-CoV-2 did not lead to local transmission while the clade G was circulating for a considerable time before the first recorded case which was of clade G and had no travel events or traveler contact^6^.

Since the declaration of COVID-19 as pandemic by the WHO on March 11th, 2020, fears were expressed that it could overwhelm weaker healthcare systems in many African countries. Furthermore, hygiene, social distancing and lockdown has caused many challenges in countries with high percentages of its citizens living without clean running water, cramped confines and the dependence on a daily income. However, Africa has the advantage of having a very young population, e.g. in Sub-Saharan Africa with a median age of 19.7 years^9^ for which on average milder etiopathologies can be expected. Additionally, for the early outbreak a study evaluating air traffic from affected regions in China calculated relatively low transmission risks for most African countries except South Africa and Ethiopia^10^. For later phases, Cabore et al. proposed a model estimating risk of exposure for African countries based on a Hidden Markov model which accounts for factors such as gathering, weather, distribution and hygiene with the conclusion that with respect to the high calculated infection rates effective containment is indispensable^11^.

Here, we analysed SARS-CoV-2 nucleotide sequences from samples obtained from the West African countries of Gambia, Ghana, Nigeria and Senegal in order to identify characteristic mutations and to dissect their patterns of distribution. The rationale of this study was to investigate commonalities and differences between SARS-CoV-2 transmission in West African countries with comparable geographic, sociological and climatic conditions. We aimed at identifying where SARS-CoV-2 was introduced into the distinct West African countries and if new potentially more dangerous variants were emerging and could be kept under surveillance. We anticipate that these results can contribute towards assessing testing strategies, establishing quarantine rules and emphasize the importance of global vaccination efforts.

## Results

### Phylogenetic tree and diversity

**Figure 1:**
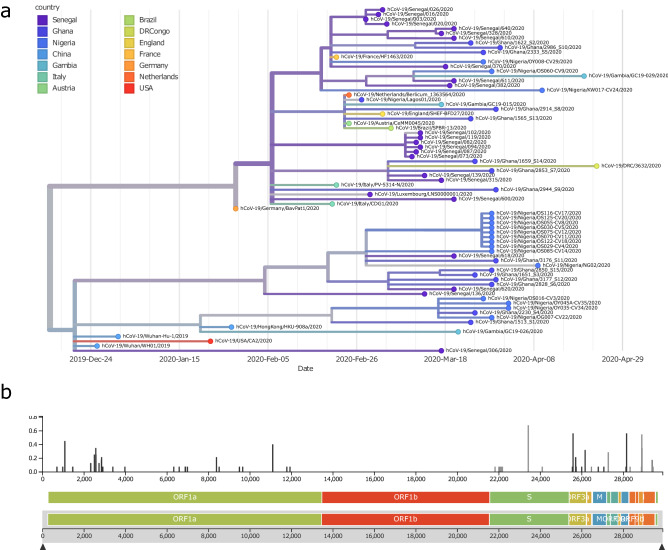
Phylogenetic tree (December 2019 to April 2020) revealing similarities of West-African viral sequences with Chinese and multiple European countries. (**a**) Nigerian samples cluster with the early Chinese samples within the bottom branch of the tree, Ghanaian samples are about equally distributed between the top (European) and bottom branch of the tree. Senegalese samples cluster closer with the French reference sample on the top of the tree. (**b**) Highest diversity is at the A23403G (D614G) substitution splitting the tree in the bottom (Chinese) and top (European) branch. This variant has been reported to increase infectivity^1,12^. Graphics were generated using the nextstrain pipeline including software Augur (version 7.0.2, https://docs.nextstrain.org/projects/augur/en/stable/index.html) and TreeTime (version 0.7.6, https://github.com/neherlab/treetime)^28,31^.

The phylogenetic tree shown in Fig. 1a displays similarities of West-African virus sequences with representative reference sequences from China and multiple European countries. The tree can be divided into two major branches resulting from the A23403G (D614G) substitution. The branch at the bottom is directly associated with the first recorded sequences from Wuhan, China and does not carry the D614G amino acid substitution. The Nigerian samples cluster with these early Chinese samples in the bottom branch of the tree. The branch on top is associated with sequences prevalent in Europe as demonstrated by reference sequences from Germany, France, Italy, Austria, Netherlands and UK. Ghanaian samples are about equally distributed between the top (European) and bottom branch of the tree. Senegalese samples cluster close with the French reference sample at the top of the tree.

The phylogenetic tree can be viewed interactively via the nextstrain.org framework under the URL: https://nextstrain.org/community/wwruck/wa.

The split of the tree by the A23403G (D614G) substitution into two major branches corresponds to the highest diversity found at that location (Fig. 1b). This mutation resides within the spike protein.

### Association with clades

**Figure 2:**
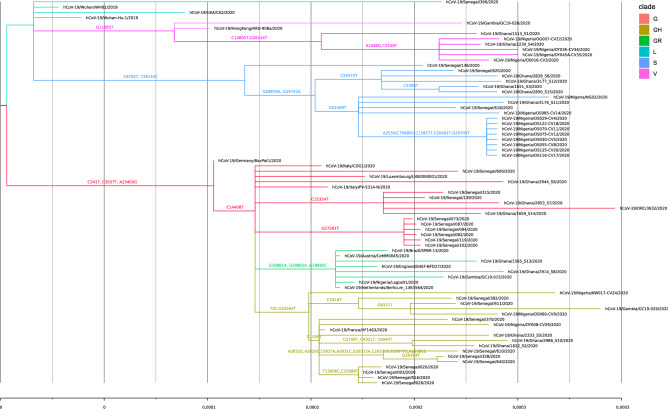
Phylogenetic tree (December 2019–April 2020) colored by clades shows distribtution of West-African samples over all clades suggesting introductions from China and European countries. Patterns are country-specific, e.g. most Senegalese samples have close similarity with the French reference, most Nigerian samples cluster in early Chinese-based clade S and Ghanaian samples are spread over all clades. Within the clade S, there are putatively specific West-African mutations at the branches at C24370T and G22468T. G22486T may reflect migration routes because in the nextstrain analysis of Africa as a whole there are also Tunisian samples in this branch (https://nextstrain.org/ncov/africa?f_region=Africa, accessed Jun 26th, 2020). Two of the non-French related Senegalese samples come from these branches while the other (Senegal/136) has strong similarity with Spanish-end of February samples from the early clade S pointing at multiple introductions to Senegal from France, Spain and African countries. Graphics were generated using the software FigTree (version 1.4.4, http://tree.bio.ed.ac.uk/software/figtree/) for annotation of the phylogenetic tree and the nextstrain pipeline including software Augur (version 7.0.2, https://docs.nextstrain.org/projects/augur/en/stable/index.html) and TreeTime (version 0.7.6, https://github.com/neherlab/treetime)^28,31^.

We associated the West-African and reference samples via their characteristic amino acid substitutions with clades according to the GISAID nomenclature. The phylogenetic tree in Fig. 2 is coloured by these clades. The West-African samples are distributed over all clades, thus suggesting introductions from China and European countries. However, each of the investigated countries has a specific pattern: most Senegalese samples have close similarity with the French reference, most Nigerian samples cluster in early Chinese-based clade S and Ghanaian samples are spread over all clades, the three Gambian samples are distributed over clades V, GR and GH. Within the clade S, there are putatively specific West-African amino acid substitutions at the branches at C24370T and G22468T. Ghanaian samples predominate in the branch associated with the C24370T mutation. The branch determined by the mutation G22486T (Supplementary Figure 1) may reflect migration routes because in the nextstrain analysis of the entire Africa there are also samples from Mali and Tunisia in this branch (https://nextstrain.org/ncov/africa?f_region=Africa, accessed August 14 2020). Two of the non-French-related Senegalese samples emanate from the C24370T and G22468T branches whilst the other (Senegal/136) has strong similarity with Spanish end-February samples from the early clade S (Supplementary Figure 2) pointing at multiple introductions to Senegal from France, Spain and African countries.

### Timeline of clade distribution

**Figure 3:**
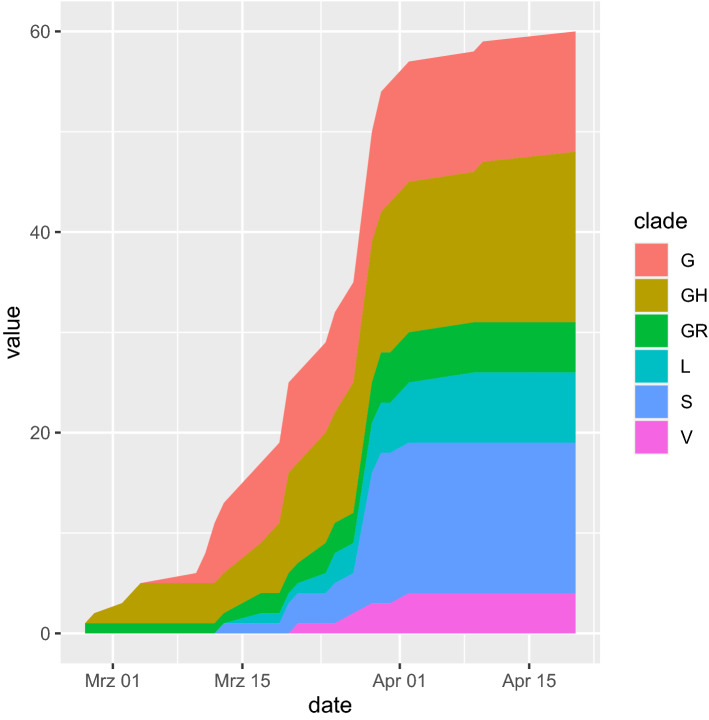
Temporal course of clade distribution confirms gaining of share of the Europe-associated G-clades harboring the putatively more infectious D614G amino acid substitution (February–April 2020). Interestingly the younger Europe-associated G-clades emerged earlier in West African sequenced samples. This could be due to founder effects by introductions from France being closely connected to Senegal and displaying a similar clade distribution. Furthermore, the China-based L-, V- and S-clade samples started in mid-March at a time when the epidemic in China was nearly entirely suppressed. Thus, the virus may have circulated in several countries before the first samples were sequenced. Surprisingly, the abundance of the S-clade is relatively high mainly due to Nigeria and Ghana but without that exception the clade distribution resembles the global one with a delay of about 2–4 weeks. The plot was generated using R (version 3.6.1, https://www.r-project.org/), [Ihaka, R. & Gentleman, R. R: A language for data analysis and graphics. *J. Comput. Graph. Stat*. **5**, 299–314 (1996)] and the R package *ggplot2* (version 3.3.0, https://ggplot2.tidyverse.org/) [Wickham, H. *Ggplot2: Elegant Graphics for Data Analysis*. (Springer, 2009).].

In the temporal course of the clade distribution in Fig. 3, the increased share of the Europe-associated G-clades becomes obvious. The G-clades harbor the putatively more infective D614G amino acid substitution^1,12^. Surprisingly, the later Europe-associated G-clades (G, GH, GR) emerged before the earlier clades L, S and V in West African sequenced samples. This could be due to founder effects introduced from France closely connected to Senegal and displaying a similar clade distribution and by migration and travel routes such as in the first registered Nigerian case infected in Italy^13^. Furthermore, the China-based L-, V- and S-clade samples were obtained in mid-March 2020, a time point within the Wuhan lockdown and when the epidemic in China was nearly over. Thus, the virus may have circulated in several countries before the first samples were sequenced. Surprisingly, the abundance of the S-clade is relatively high mainly due to the contribution from Nigeria and Ghana. However, without the S-clade distribution, the change in abundance resembles the global one with a delay of about 2–4 weeks.

### Country-specific patterns of clade distribution

**Figure 4:**
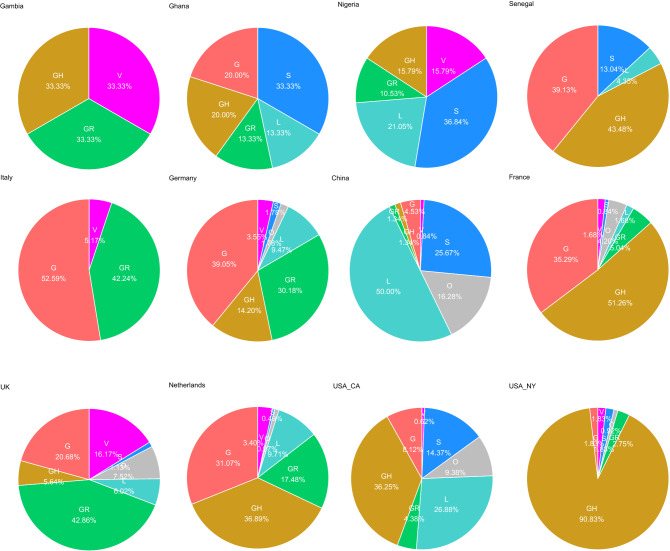
West African countries display distinct patterns of China-and Europe-based clades (until April 2020). Nigeria has the highest percentage of the China-based early clades (L, S, V) and Ghana has nearly equally distributed percentages of China and Europe-based clades (G, GH, GR). Senegal has a similar clade distribution as France but also a few samples from the early China-based clades. In Gambia there were only three sequences, two from Europe-based clades GR and GH and one from China-based clade V. The charts were generated using R (version 3.6.1, https://www.r-project.org/), [Ihaka, R. & Gentleman, R. R: A language for data analysis and graphics. *J. Comput. Graph. Stat*. **5**, 299–314 (1996)] and the R package *ggplot2* (version 3.3.0, https://ggplot2.tidyverse.org/), [Wickham, H. *Ggplot2: Elegant Graphics for Data Analysis*. (Springer, 2009).].

Figure 4 shows that West African countries have acquired distinct patterns of China-and Europe-based clades. The first row contains the clade distribution charts of the West African countries investigated here whilst the second row contains charts of countries with comparable distributions. Nigeria has the highest percentage of the China-based early clades (L, S, V). Ghana has nearly equally distributed percentages of China and Europe-based clades (G, GH, GR) and in that sense has similarities with the German distribution. Senegal’s clade distribution resembles the one from France but includes also a few samples from the early China-based clades. There were only three sequences from Gambian, two from Europe-based clades GR and GH and one from China-based clade V. That pattern resembles the one from Italy when the clade G is substituted by the G-derived GH clade which however does not infer a connection to Italy but instead a similar combination of Chinese and European-related clades. Also the UK distribution in the last row shares similarities with the Gambian distribution but as it also includes Chinese clades it also resemble the one from Ghana. The Dutch distribution which is quite similar to the German also resembles the clade distribution from Ghana. Last but not least, there are the quite distinct distribution from the US West and East Coast (California, CA and New York, NY). The Californian chart has similarity with the Nigerian because of the high percentage of Chinese-based clades while the chart from New York has a comparable high percentage of clade GH as the ones from France and Senegal.

### Geographic distribution

**Figure 5:**
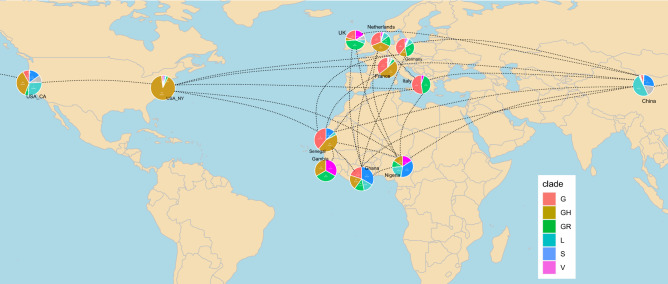
Geographic map reveals distinct patterns of introduction of China-and Europe-based clades in West African countries (until April 2020). Nigeria with the highest percentage of the China-based early clades (L, S, V) and Ghana with nearly equally distributed percentages of China and Europe-based clades (G, GH, GR) might be comparable with the US West Coast while Senegal with a similar clade distribution like France and few samples from the early China-based clades may be more comparable to the US East Coast. It will be interesting to observe if the later G clades replace the early clades in Nigeria and Ghana and if that correlates with the severity of the disease as was postulated for the US. The R package *rworldmap* (version 1.3-6, https://cran.r-project.org/web/packages/rworldmap/index.html), South^32^ was used to generate the world map in this figure using R (version 3.6.1, https://www.r-project.org/), [Ihaka, R. & Gentleman, R. R: A language for data analysis and graphics. *J. Comput. Graph. Stat*. **5**, 299–314 (1996)].

The world map in Fig. 5 reveals the distinct combinations of introduction of China-and Europe-based clades in West African countries. Nigeria has the highest percentage of the early clades (L, S, V) which were based in China but subsequently distributed to Europe and to the West Coast of USA. Ghana possesses nearly equally distributed percentages of the early clades and the Europe-based clades (G, GH, GR) comparable to the West Coast of USA. Senegal has a similar clade distribution like France and only few samples from the early China-based clades might be more comparable to the US East Coast.

We set out to further explore the above-mentioned surprising observation (Fig. 3) that in West Africa the early clades emerged after the later Europe-associated G-clades. Possible explanations could be (1) latent circulation of the early clades in West Africa or (2) later introduction of the earlier clades. With the aim to find evidence for one of these alternatives, we looked into detail of the phylogeny of samples from the earlier clades. We picked two samples from the early clades: sample Senegal/136/2020 comes from a phylogenetic branch predominated by Spanish samples but also including samples from Asia and Latin America (Suppl. Figure 2), several West African samples from Nigeria (dated March 29th, 2020), Ghana and Senegal in the phylogenetic branch in Suppl. Figure 3 have a long latency time of about 2 months to the estimated common predecessor estimated on January 29th, 2020. Thus, there is evidence for a combination of both explanations: SARS-CoV-2 samples of the early clades may have circulated latently in West Africa since January 2020 but additionally there might have been introductions of the early clades from Europe and Asia or via maritime trade.

### Emergence of new variants from July 2020 to June 2021

**Figure 6:**
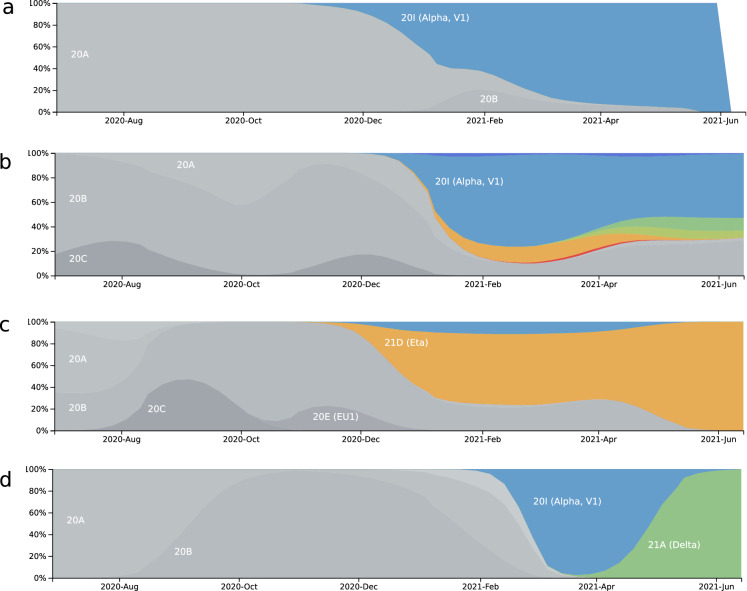
Distribution plot of SARS-CoV-2 variants from July 2020 to June 2021 shows distinct patterns in West African countries. This plot was generated in June 2021 and confirms our previous analysis of samples from February 2020 to April 2020 predicting a rapid spread of the variants carrying the D614G amino acid substitution. All predominating variants in June 2021 also carried the D614G, besides their characteristic amino acid changes. (**a**) Gambia is dominated by the Alpha variant. (**b**) Ghana has a combination of the variants Alpha, Delta, Kappa, Eta and Iota, dominated by the Alpha. (**c**) Nigeria is dominated by the Eta variant (B.1.525) which putatively originated from Nigeria. (**d**) In Senegal the Delta variant displaced the Alpha variant. Variants are marked by the colors blue (Alpha), green (Delta), yellow green (Kappa), orange (Eta) and red (Iota). Figure adapted from the website www.nextstrain.org (accessed June 22, 2021, CC-BY^28,31^).

As in parallel with the review process several new variants had emerged we updated the distribution of new variants in June 2021. The SARS-CoV-2 variant distribution plots in Fig. 6 reveal distinct patterns of variants predominantly related to introductions from Europe but also the Eta variant (B.1.525) which is of Nigerian origin^14,15^. Gambia (Fig. 6a) is dominated by the Alpha variant (B.1.1.7) which originated from the UK and is also predominant in Ghana (Fig. 6b). However, similar to the analysis from 2020 (Fig. 3) Ghana has the most diverse combination of variants, also including Delta, Kappa, Eta and Iota, thus reflecting putative introductions from Europe, India (Delta and Kappa probably via Europe), US (Iota) and Nigeria (Eta). Nigeria (Fig. 6c) is dominated by the Eta variant which has been described to have originated from within. Senegal (Fig. 6d) was initially dominated by the Alpha variant but later redominated by the Delta variant (B.1.617.2), this is expected to happen in many European countries. Interestingly, in Ghana (Fig. 6b) the Delta variant did not replace the Alpha and other variants until the end of June 2021 probably due to a latent circulation.

Most of the variants found in the distribution plots were updated in June 2021, unfortunately recent information about quantitative features is scarce. However, in a technical briefing from Public Health England from June 2021, case-fatality-rates (among those with 28 day follow up) for relevant variants were calculated for England between February 1st, 2021 and June 21st, 2021^16^: 1.9% (Alpha), 0.3% (Delta) and 2.8% (Eta), corresponding case proportions were: 70.3% (Alpha), 28.8% (Delta) and 0.1% (Eta). For the interpretation of these rates, apparently the progress of the vaccination campaign has to be taken into account with the dynamics of the pandemic: alpha started in about October 2020, reached 100% proportion in March 2021 and was displaced by Delta around April 2021 and peaked at 94% in June 2021. Thus, the late emergence of the Delta variant coinciding with a large percentage of vaccinated people may largely contribute to the low case-fatality-rate of the Delta variant. For comparison, in Pune India with a much lower percentage of vaccinated people compared to the UK, a case-fatality rate of 1.1% was announced for the Delta variant^17^. Zani et al*.* reported in a serosurvey of BNT162b2 vaccine-elicited neutralizing antibodies, that vaccination was more effective on Alpha and Eta than in the Beta (B.1.351) and Gamma (P.1) variants^18^. Campbell et al*.* calculated that the Eta and Alpha variant relative to non-variants had about a 25% higher effective reproducibility while the Delta variant had nearly a 100% higher effective reproducibility^19^.

## Discussion

In this phylogenetic analysis of SARS-CoV-2 sequences from the West African countries Gambia, Ghana, Nigeria and Senegal, we identified country-specific patterns of earlier (L, S, V) and later Europe-associated (G, GR, GH) clades. In Senegal and Gambia, the later Europe-associated clades were predominant, in Ghana earlier and later clades were more equally distributed and in Nigeria the earlier clades were the predominant samples downloaded from the GISAID database in June 2020. This would suggest multiple introductions mainly from Europe into Senegal and Gambia, from Europe and directly or indirectly via other Asian or European countries from China then to Ghana and Nigeria. The introductions from China to Nigeria and Ghana are in line with a study by Haider et al*.* in which both countries, but not Senegal and Gambia, appear in a table of estimations of SARS-CoV-2 transmission risk from China based on air traffic statistics^10^. However, they are at low risk in the second quartile—with the fourth quartile having the highest risk. There was a lack of data for Senegal and Gambia therefore hinting to no or only low-level direct air traffic connection to China, thus suggesting a predominant introduction from Europe.

Against our expectations, we found that the later European-associated clades (G, GR, GH) emerged before the earlier Chinese-based clades (L, S, V) in the registered cases in the investigated West African countries. We propose the following hypothesis as an explanation to this surprising observation: the early clades were already circulating within the populations before the later European-associated clades were introduced. A higher disease severity of the later European clades might then be a possible explanation for their earlier detection. Intriguingly, most of the cases investigated in this study occurred within the time interval of the Wuhan lockdown between January 23rd and April 8th, 2020. Thus, transmission of the early clades must have taken place very early or via intermediate countries or other Chinese provinces. Besides the later Europe-associated G-clades, the early clades were also circulating in Europe and the US West coast of USA, for example, the Senegal sample no. 136 from the early clade S bears similarity with Spanish samples (Suppl. Figure 2). Other explanations for the relatively long latency may be founder effects that by chance individuals infected with the later clades travelled to West Africa before individuals infected with the earlier clades—or slower means of transportation such as ships commuting between China, America, Europe and West Africa.

Based on previous reports^1^, it might probably be that the later G clades will replace the early clades in Nigeria and Ghana. The question if that correlates with the severity of the disease still needs to be addressed, Brufsky infers it from the higher mortality at the East Coast of USA with predominantly D614G-carrying G-clades compared to the West Coast with the predominant early clades^2^. Becerra-Flores et al. found significant correlations between the percentage of D614G and case-fatality on a country by country basis^20^. However, others find evidence for higher transmissibility and also higher viral-load but no evidence for higher disease severity^1,21,22^. A correlation of the amino acid substituted D614G associated with the G-clades and case fatality in the West African countries can only be identified at a marginal level of r = 0.28 (Supplementary Table 1). The case fatality is fortunately rather low ranging from 0.6 in Ghana up to 3.2 in Gambia. Other factors such as climate, sunlight exposure^23^ and associated Vitamin D^24^, medical infrastructure and demographics might influence the etiopathology even more. There are also perspectives of decreased disease severity as Benedetti et al*.* argue that SARS-CoV-2 will mutate continuously and attenuate naturally to become endemic at a low mortality rate^25^, as has been observed with earlier viruses^26^.

The limitations of this study are the sample size, possible selection bias of the samples and the intrinsic incompleteness of the phylogenetic analysis which may lead to altered results when more samples are included. Nonetheless, this is the first study of its kind, the data and concept should form the basis for a more extensive analysis due to an increased number of sequenced samples becoming available.

Supplementary Figure 4 shows the positive rates and total tests performed in the West African countries without and with reference curves from Switzerland, a country which applied no extreme strategies to manage the pandemic and had incidence values ranging at about the average of European countries. Senegal and Gambia display a high positive rate going up to 50%, while Nigeria and Ghana have peaks at about 30% comparable to Switzerland. However, Nigeria has the lowest and Ghana the highest numbers of tests performed of the four countries. Switzerland started testing much later than the four West African countries but then tested at a higher rate. For the analysis of samples from February 2020 to April 2020 there were three sequence samples from Gambia, 15 from Ghana, 19 from Nigeria and 23 from Senegal available from West Africa. The variant distribution plots from June 2021 were based on 30 sequence samples from Gambia, 130 from Ghana, 127 from Nigeria and 41 from Senegal. The high positive rates of tests and in addition the low numbers of samples sequenced suggest a high number of undetected infections and are in line with latent circulation of new variants. Although the diversity of variants found demonstrates a certain degree of representativeness we have to state that one limitation of this study may be selection bias.

The distinct distribution plots of SARS-CoV-2 variants in the West African countries showed that there may be more virus transmission via air traffic predominantly from Europe than via regional traffic between the West African countries themselves. Indications for this are limited to marginal occurrence of the putatively Nigerian Eta variant^14,15^ in Ghana and to common occurrences of the Alpha variant in Senegal, Gambia and Ghana. Together with the partially high test-positive rates and implicated high number of undetected cases and potentially undetected new variants these results show a dynamic pandemic progression. The D614G amino acid substitution which was the most relevant change in SARS-CoV-2 in spring 2020 became prevalent in 2021 and was boosted by continuously acquired new mutations agglomerating into new variants. Fortunately, there are vaccines which are effective against new variants, and even against the new putative Nigerian Eta variant^18,27^ but in the long-term evasive variants may develop if the transmission of Covid19 is not effectively halted.. This underscores the importance of thorough surveillance of infections, new variants and global vaccination efforts.

In conclusion, in this phylogenetic analysis of SARS-CoV-2, we found distinct patterns of viral variants, until April 2020 the later Europe-associated G-clades were predominant in Senegal and Gambia, and combinations of the earlier (L, S, V) and later clades in Ghana and Nigeria. Intriguingly, the later clades emerged before the earlier clades which could simply be due to founder effects or due to latent circulation of the earlier clades. In June 2021, again introductions from Europe were predominant in Gambia (Alpha) and Senegal (Alpha and Delta from India via Europe), a combination of Europe-associated and others in Ghana and a putatively Nigerian-originating Eta variant in Nigeria. Only a marginal correlation of the G-clades in the West African countries until April 2020 can be associated with mortality which fortunately is at a rather low level therefore disproving fears that the pandemic would massively overwhelm the health systems in Africa. The rather young population and the climate might be factors favoring this low infectivity and fatality rate in comparison to Western countries but nevertheless a cautious balance between health protection and economics might prevent future disastrous outbreaks.

## Methods

### Sample collection

We downloaded SARS-CoV-2 viral sequences for West African samples and reference samples from European, North and South American countries and China from the GISAID database of June 2020. The samples used in this study are shown in Table 1.

**Table 1:** SARS-CoV-2samples used for the phylogenetic analysis.

Region	Name	Accession	Date	Clade
West Africa	hCoV-19/Gambia/GC19-029/2020	EPI_ISL_428857	4/20/2020	GH
	hCoV-19/Senegal/611/2020	EPI_ISL_420076	3/20/2020	GH
	hCoV-19/Senegal/003/2020	EPI_ISL_418206	2/28/2020	GH
	hCoV-19/Senegal/016/2020	EPI_ISL_418207	3/2/2020	GH
	hCoV-19/Senegal/026/2020	EPI_ISL_418209	3/3/2020	GH
	hCoV-19/Senegal/020/2020	EPI_ISL_418208	3/4/2020	GH
	hCoV-19/Senegal/382/2020	EPI_ISL_420073	3/19/2020	GH
	hCoV-19/Senegal/370/2020	EPI_ISL_420072	3/18/2020	GH
	hCoV-19/Nigeria/OY008-CV29/2020	EPI_ISL_455429	3/27/2020	GH
	hCoV-19/Nigeria/KW017-CV24/2020	EPI_ISL_455362	4/10/2020	GH
	hCoV-19/Ghana/2333_S5/2020	EPI_ISL_422394	3/27/2020	GH
	hCoV-19/Ghana/2986_S10/2020	EPI_ISL_422401	3/31/2020	GH
	hCoV-19/Ghana/1622_S2/2020	EPI_ISL_422384	3/24/2020	GH
	hCoV-19/Nigeria/OS060-CV9/2020	EPI_ISL_455419	3/29/2020	GH
	hCoV-19/Senegal/610/2020	EPI_ISL_420075	3/20/2020	GH
	hCoV-19/Senegal/328/2020	EPI_ISL_420071	3/17/2020	GH
	hCoV-19/Senegal/640/2020	EPI_ISL_420079	3/20/2020	GH
	hCoV-19/Gambia/GC19-015/2020	EPI_ISL_428855	3/17/2020	GR
	hCoV-19/Ghana/2914_S8/2020	EPI_ISL_422399	3/30/2020	GR
	hCoV-19/Nigeria/NG57752/2020	EPI_ISL_462992	2020-03	GR
	hCoV-19/Ghana/1565_S13/2020	EPI_ISL_422404	3/24/2020	GR
	hCoV-19/Nigeria/Lagos01/2020	EPI_ISL_413550	2/27/2020	GR
	hCoV-19/Senegal/315/2020	EPI_ISL_420070	3/17/2020	G
	hCoV-19/Senegal/600/2020	EPI_ISL_420074	3/20/2020	G
	hCoV-19/Senegal/073/2020	EPI_ISL_418210	3/10/2020	G
	hCoV-19/Senegal/082/2020	EPI_ISL_418211	3/11/2020	G
	hCoV-19/Senegal/087/2020	EPI_ISL_418212	3/11/2020	G
	hCoV-19/Senegal/094/2020	EPI_ISL_418213	3/12/2020	G
	hCoV-19/Senegal/119/2020	EPI_ISL_418215	3/12/2020	G
	hCoV-19/Ghana/2853_S7/2020	EPI_ISL_422398	3/29/2020	G
	hCoV-19/Senegal/139/2020	EPI_ISL_418217	3/13/2020	G
	hCoV-19/Ghana/2944_S9/2020	EPI_ISL_422400	3/30/2020	G
	hCoV-19/Ghana/1659_S14/2020	EPI_ISL_422405	3/25/2020	G
	hCoV-19/Senegal/102/2020	EPI_ISL_418214	3/12/2020	G
	hCoV-19/Senegal/618/2020	EPI_ISL_420077	3/20/2020	S
	hCoV-19/Senegal/136/2020	EPI_ISL_418216	3/13/2020	S
	hCoV-19/Senegal/620/2020	EPI_ISL_420078	3/20/2020	S
	hCoV-19/Ghana/1651_S3/2020	EPI_ISL_422387	3/25/2020	S
	hCoV-19/Ghana/2850_S15/2020	EPI_ISL_422406	3/29/2020	S
	hCoV-19/Ghana/2828_S6/2020	EPI_ISL_422397	3/29/2020	S
	hCoV-19/Ghana/3177_S12/2020	EPI_ISL_422403	3/30/2020	S
	hCoV-19/Ghana/3176_S11/2020	EPI_ISL_422402	3/30/2020	S
	hCoV-19/Nigeria/OS030-CV5/2020	EPI_ISL_455415	3/29/2020	S
	hCoV-19/Nigeria/OS085-CV14/2020	EPI_ISL_455424	3/29/2020	S
	hCoV-19/Nigeria/OS055-CV8/2020	EPI_ISL_455418	3/29/2020	S
	hCoV-19/Nigeria/OS070-CV11/2020	EPI_ISL_455422	3/29/2020	S
	hCoV-19/Nigeria/OS075-CV12/2020	EPI_ISL_455423	3/29/2020	S
	hCoV-19/Nigeria/OS122-CV18/2020	EPI_ISL_455426	3/29/2020	S
	hCoV-19/Nigeria/OS125-CV20/2020	EPI_ISL_455427	3/29/2020	S
	hCoV-19/Gambia/GC19-026/2020	EPI_ISL_428856	3/21/2020	V
	hCoV-19/Nigeria/OG007-CV22/2020	EPI_ISL_455412	3/29/2020	V
	hCoV-19/Nigeria/OY045A-CV35/2020	EPI_ISL_455431	4/2/2020	V
	hCoV-19/Nigeria/OS016-CV3/2020	EPI_ISL_455413	3/27/2020	V
	hCoV-19/Senegal/306/2020	EPI_ISL_420069	3/17/2020	L
	hCoV-19/Ghana/2230_S4/2020	EPI_ISL_422390	3/25/2020	L
	hCoV-19/Nigeria/OY035-CV34/2020	EPI_ISL_455430	4/2/2020	L
	hCoV-19/Ghana/1513_S1/2020	EPI_ISL_422382	3/24/2020	L
	hCoV-19/Nigeria/OS029-CV4/2020	EPI_ISL_455414	3/29/2020	L
	hCoV-19/Nigeria/OS116-CV17/2020	EPI_ISL_455425	3/29/2020	L
	hCoV-19/Nigeria/NG02/2020	EPI_ISL_450507	4/9/2020	L
Others	hCoV-19/France/HF1463/2020	EPI_ISL_429968	2/21/2020	GH
	hCoV-19/England/SHEF-BFD27/2020	EPI_ISL_416737	3/3/2020	GR
	hCoV-19/Brazil/SPBR-13/2020	EPI_ISL_416035	3/5/2020	GR
	hCoV-19/Austria/CeMM0045/2020	EPI_ISL_437932	2/24/2020	GR
	hCoV-19/Netherlands/Berlicum_1363564/2020	EPI_ISL_413565	2/24/2020	GR
	hCoV-19/Italy/CDG1/2020	EPI_ISL_412973	2/20/2020	G
	hCoV-19/Germany/BavPat1/2020	EPI_ISL_406862	1/28/2020	G
	hCoV-19/Italy/PV-5314-N/2020	EPI_ISL_451307	2/21/2020	G
	hCoV-19/Luxembourg/LNS0000001/2020	EPI_ISL_419562	2/29/2020	G
	hCoV-19/DRC/3632/2020	EPI_ISL_447607	4/23/2020	G
	hCoV-19/HongKong/HKU-908a/2020	EPI_ISL_434569	1/27/2020	V
	hCoV-19/DRC/3632/2020	EPI_ISL_447607	4/23/2020	V
	hCoV-19/Wuhan-Hu-1/2019	EPI_ISL_402125	12/31/2019	L
	hCoV-19/USA/CA2/2020	EPI_ISL_406036	1/22/2020	L
	hCoV-19/Wuhan/WH01/2019	EPI_ISL_406798	12/26/2019	L

### Construction of the phylogenetic tree

The phylogenetic tree was constructed using a pipeline adapted from the Zika virus pipeline on the nextstrain.org web page^28^ employing the Augur^28^, the MAFFT^29^ and the IQ-tree^30^ software. Details of steps which were performed:

First all West African and reference sequences in FASTA format were aligned employing the augur command:

- *augur align –sequences westafrica.fasta –reference-sequence sars_cov2_referencesequence.gb –output wa_aligned.fasta –fill-gaps.*

which called MAFFT^29^ with the command:

- *mafft –reorder –anysymbol –nomemsave –adjustdirection –thread 1 wa_aligned.fasta.to_align.fasta 1* > *wa_aligned.fasta 2* > *wa_aligned.fasta.log.*

Metadata was extracted from the sequences FASTA via the augur command:

- *augur parse –sequences wa_aligned.fasta –fields strain accession date –output-sequences wa_aligned_parsed.fasta –output-metadata metadata.tsv.*

Then a tree was built via the augur command:

- *augur tree –alignment wa_aligned_parsed.fasta –output wa_tree_raw.nwk*

Calling the IQ-tree algorithm^30^ via this command:

- *iqtree -ninit 2 -n 2 -me 0.05 -nt 1 -s wa_aligned_parsed-delim.fasta -m GTR* > *wa_aligned_parsed-delim.iqtree.log*

The tree was refined via the Augur software calling TreeTime^31^ for Maximum-Likelihood analysis inferring a time resolved phylogeny tree:

- *augur refine –tree wa_tree_raw.nwk –alignment wa_aligned_parsed.fasta –metadata metadata.tsv –output-tree wa_tree.nwk –output-node-data wa_branch_lengths.json –timetree –coalescent opt –date-confidence –date-inference marginal –clock-filter-iqd 4 –keep-polytomies.*

Here, the command from the Zika pieline was adapted to –keep-polytomies to keep all samples.

Metadata information was manually supplemented with country and region information and associated with the tree via a call to Augur:

- *augur traits –tree wa_tree.nwk –metadata metadata_countries.tsv –output wa_traits.json –columns region country –confidence.*

Augur was called to infer ancestral states of discrete character again using TreeTime^31^:

- *augur ancestral –tree wa_tree.nwk –alignment wa_aligned_parsed.fasta –output-node-data wa_nt_muts.json –inference joint.*

Amino acid changes were identified with the augur translate command:

- *augur translate –tree wa_tree.nwk –ancestral-sequences wa_nt_muts.json –reference-sequence sars_cov2_referencesequence.gb –output wa_aa_muts.json.*

Results were exported via the augur command:

- *augur export v2 –tree wa_tree.nwk –metadata metadata_countries.tsv –node-data wa_branch_lengths.json wa_traits.json wa_nt_muts.json wa_aa_muts.json –colors colors.tsv –lat-longs lat_longs.tsv –auspice-config auspice_config.json –output wa_cov19.json.*

### Visualization of the phylogenetic tree and annotation with mutations and clades

The phylogenetic tree was annotated with crucial mutations using the tool FigTree version 1.4.4 (http://tree.bio.ed.ac.uk/software/figtree/). Branches of the tree corresponding to clades following the nomenclature of GISAID were coloured distinctly.

### World map chart

The world map chart was built using the R-package *rworldmap*^32^. Clade distribution pie charts were copied to the distinct country locations. Connections between countries were based on the nextstrain Africa analysis and our own auspice analysis. Further connections between countries were retrieved from literature on virus introductions into countries or regions. The first patient on the West Coast of the United States returned from a journey to Wuhan, China^33^. The first introductions in New York came from multiple independent infected individuals mainly from Europe^7^. The first cases in France and Europe were Chinese travelers from the predominantly affected Hubei province who entered the country in mid-January and were tested positive on January 24th, 2020^34^. Patient zero in Germany was a Chinese resident from Wuhan visiting Germany^35^. The Italian outbreak started with two Chinese travelers who arrived in Milan-Lombardy, went to Rome later on and were tested positive on January 31st, 2020^36^. The first Italian citizen was confirmed positive for COVID-19 on February 21st, 2020 in Lombardy^36^. In the Netherlands, the first patient diagnosed on February 27th, 2020 had probably infected himself on a trip to Northern Italy between February 18th and 21st^37^. The first cases in the UK returned from travels to the Chinese Hubei province and were tested positive for SARS-CoV-2 on January 30th, 2020^38^.

### June 2021 update of the variant distribution and test-positive- rate plots

Variant distribution plots were downloaded from the website nextstrain.org^28,31^ on June 22nd, 2021 filtering for Africa and the distinct countries Gambia, Ghana, Nigeria and Senegal. Plots of test-positive-rates and tests per thousand people were downloaded from the website OurWorldInData.org/coronavirus^39^ selecting the countries Gambia, Ghana, Nigeria and Senegal for both plots. Data from Switzerland, a country which had neither an extreme pandemic management strategy nor an extreme outbreak, was integrated as reference in additional plots.

## Supplementary Information

Supplementary Legends.

Supplementary Figure 1.

Supplementary Figure 2.

Supplementary Figure 3.

Supplementary Figure 4.

Supplementary Table 1.

Supplementary Table 2.
