# Supplementary material for: Detailed phylogenetic analysis tracks transmission of distinct SARS-COV-2 variants from China and Europe to West Africa
Source: Sci Rep. 2021 Oct 26;11:21108. doi: 10.1038/s41598-021-00267-w (PMC8548492; doi:10.1038/s41598-021-00267-w)
Supplement: Supplementary file 1 — Supplementary Legends. [file 41598_2021_267_MOESM1_ESM.docx]

**Supplementary Material for the article “****Detailed phylogenetic analysis tracks transmission of distinct SARS-COV-2 variants from China and Europe to West Africa.”**

Wasco Wruck^1^ and James Adjaye^1,*^

^1^Institute for Stem Cell Research and Regenerative Medicine, Medical Faculty, Heinrich Heine University, 40225 Düsseldorf, Germany

^*^corresponding author: James Adjaye ([james.adjaye@med.uni-duesseldorf.de](mailto:james.adjaye@med.uni-duesseldorf.de))

**Supplementary Table 1: Case fatality and percentage of mutation D614G in West African countries**

**Supplementary Table 2: Acknowledgement table of sequence samples from the GISAID database**

**Supplementary Figure 1:** **Detailed phylogenetic analysis of the Senegal/618 and several Nigerian samples point at introduction through travel or migration routes via Tunisia, Egypt and Mali.**

**Supplementary Figure 2:** **Detailed phylogenetic analysis of the Senegal/136 sample suggests introduction from Spain.**

**Supplementary Figure 3:** **Detailed phylogenetic analysis of Nigerian, Ghanaian and Senegalese samples points at long latent circulation of early clades of SARS-CoV-2 in these countries between end of January until end of March 2020.**

**Supplementary Figure 4:** **Positive rates and numbers of tests for SARS-CoV-2 in Gambia, Ghana, Nigeria and Senegal**.(a) share of positive COVID-19 tests, (b) share of positive COVID-19 tests including Switzerland as reference, (c) daily COVID-19 tests per thousand people, (d) daily COVID-19 tests per thousand people including Switzerland as reference. (Figure adapted from the website OurWorldInData.org/coronavirus (accessed June 22 2021, CC-BY, [Hasell, J. et al. A cross-country database of COVID-19 testing. Sci. Data 7, 345 (2020).]).
