# Supplementary figures and images for: Detailed phylogenetic analysis tracks transmission of distinct SARS-COV-2 variants from China and Europe to West Africa
Source: Sci Rep. 2021 Oct 26;11:21108. doi: 10.1038/s41598-021-00267-w (PMC8548492; doi:10.1038/s41598-021-00267-w)

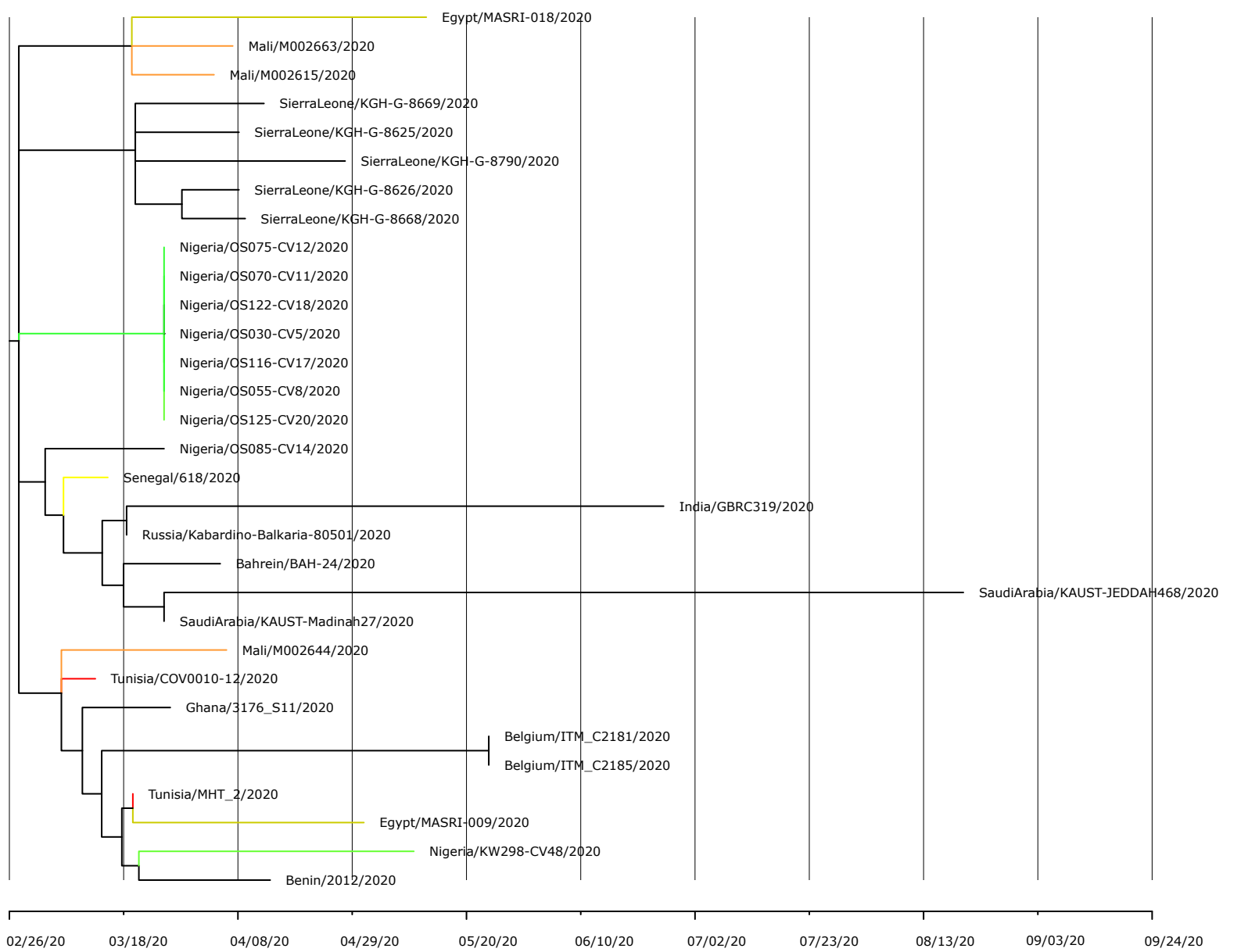

Supplement: Supplementary file 2 — Supplementary Figure 1. [file 41598_2021_267_MOESM2_ESM.pdf]

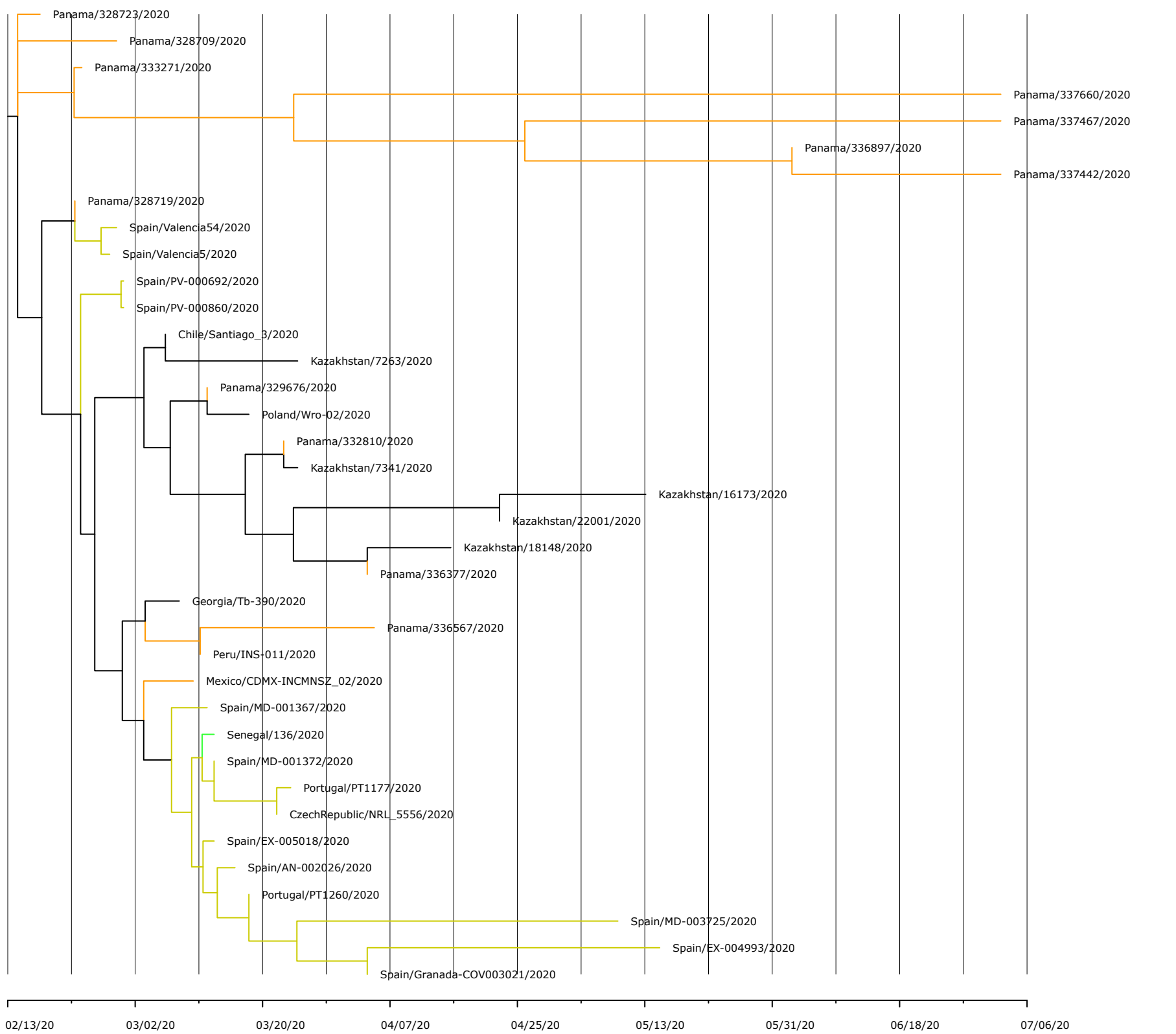

Supplement: Supplementary file 3 — Supplementary Figure 2. [file 41598_2021_267_MOESM3_ESM.pdf]

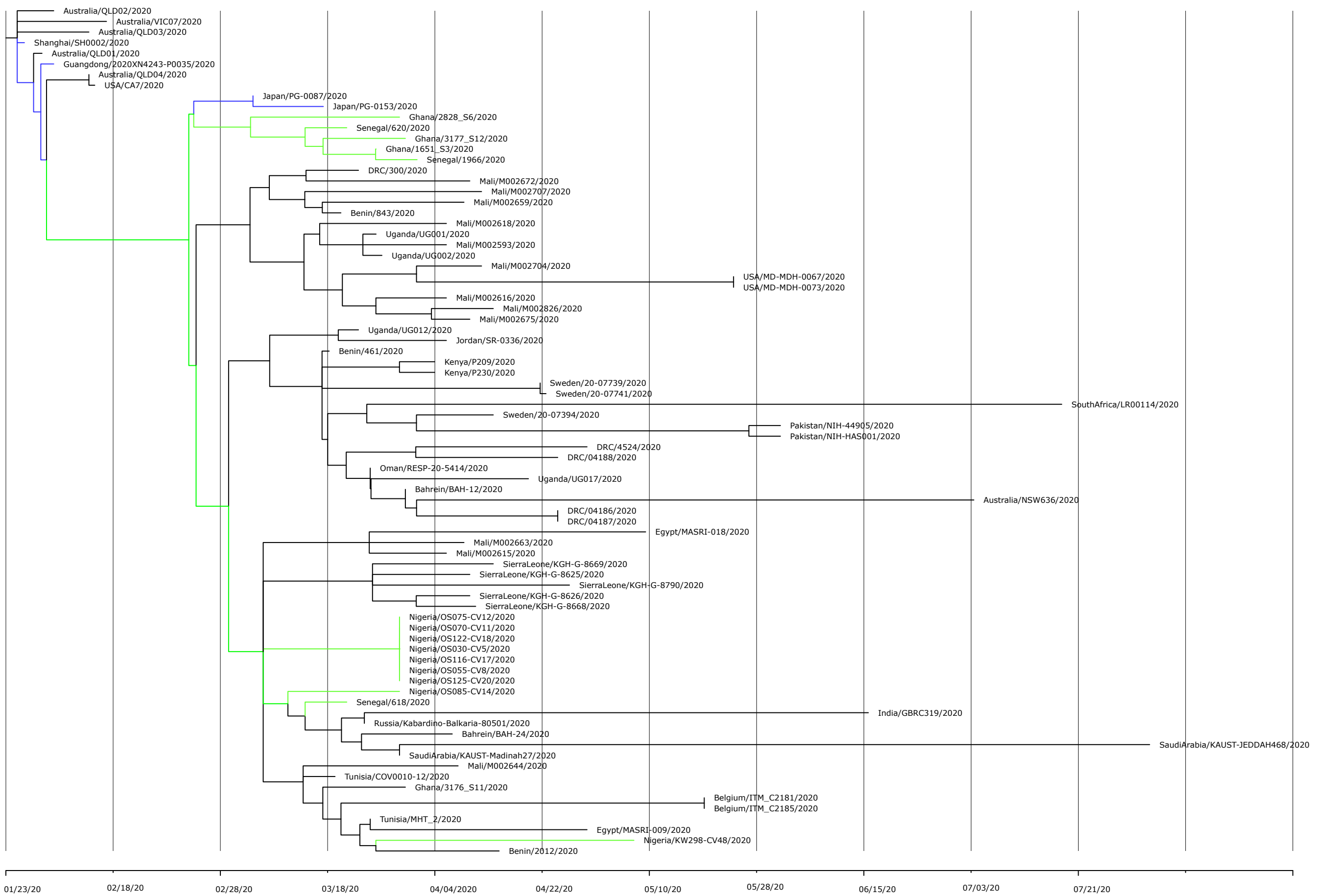

Supplement: Supplementary file 4 — Supplementary Figure 3. [file 41598_2021_267_MOESM4_ESM.pdf]
