# Supplementary material for: Detailed phylogenetic analysis tracks transmission of distinct SARS-COV-2 variants from China and Europe to West Africa
Source: Sci Rep. 2021 Oct 26;11:21108. doi: 10.1038/s41598-021-00267-w (PMC8548492; doi:10.1038/s41598-021-00267-w)
Supplement: Supplementary file 5 — Supplementary Figure 4. [file 41598_2021_267_MOESM5_ESM.pdf]

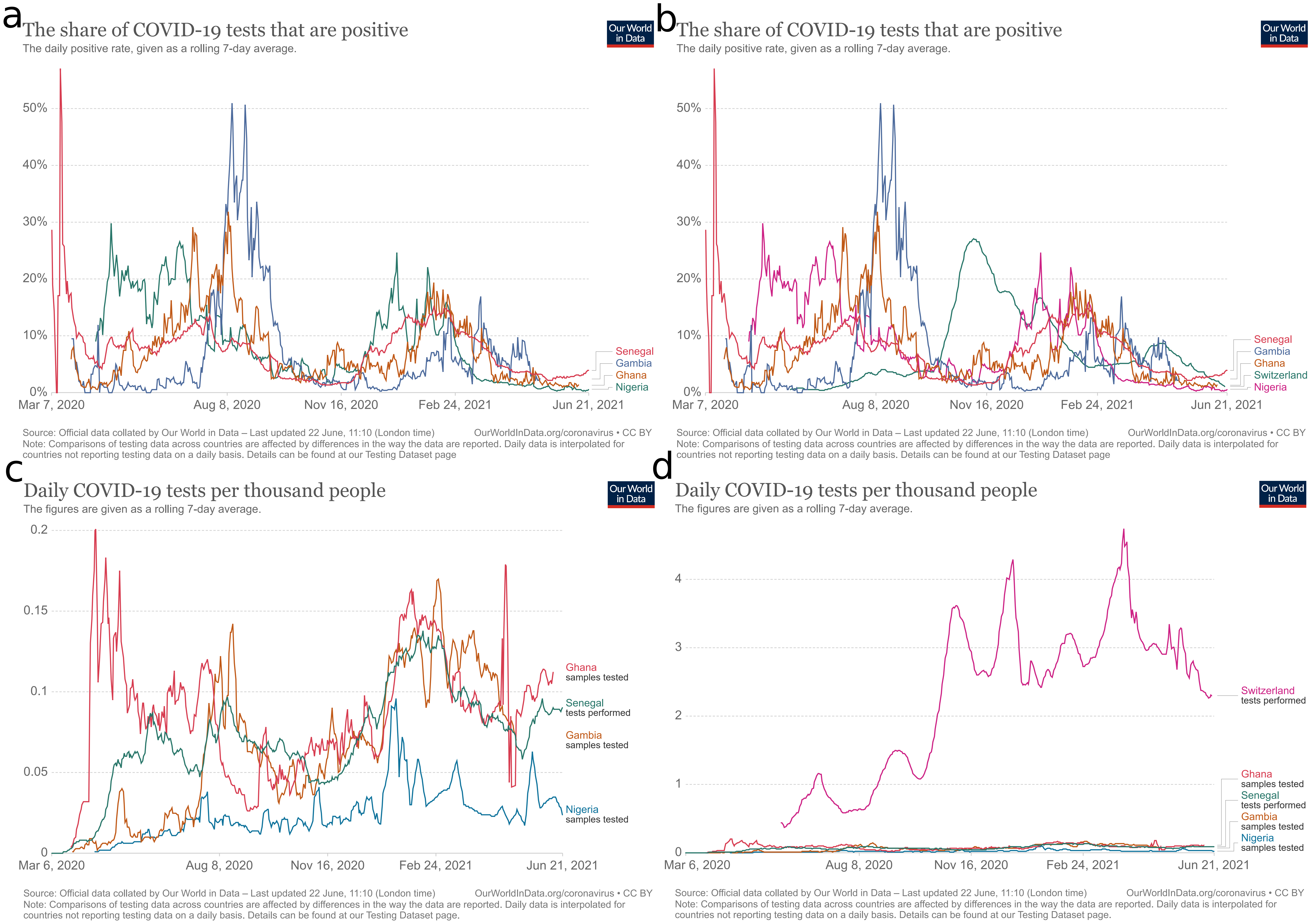
a
The share of COVID-19 tests that are positive
The daily positive rate, given as a rolling 7-day average.
Our World in Data
50%
40%
30%
20%
10%
0%
Mar 7, 2020
Aug 8, 2020
Nov 16, 2020
Feb 24, 2021
Jun 21, 2021
Senegal
Gambia
Ghana
Nigeria
Source: Official data collated by Our World in Data – Last updated 22 June, 11:10 (London time)
OurWorldInData.org/coronavirus • CC BY
Note: Comparisons of testing data across countries are affected by differences in the way the data are reported. Daily data is interpolated for countries not reporting testing data on a daily basis. Details can be found at our Testing Dataset page
b
The share of COVID-19 tests that are positive
The daily positive rate, given as a rolling 7-day average.
Our World in Data
50%
40%
30%
20%
10%
0%
Mar 7, 2020
Aug 8, 2020
Nov 16, 2020
Feb 24, 2021
Jun 21, 2021
Senegal
Gambia
Ghana
Switzerland
Nigeria
Source: Official data collated by Our World in Data – Last updated 22 June, 11:10 (London time)
OurWorldInData.org/coronavirus • CC BY
Note: Comparisons of testing data across countries are affected by differences in the way the data are reported. Daily data is interpolated for countries not reporting testing data on a daily basis. Details can be found at our Testing Dataset page
c
Daily COVID-19 tests per thousand people
The figures are given as a rolling 7-day average.
Our World in Data
0.2
0.15
0.1
0.05
0
Mar 6, 2020
Aug 8, 2020
Nov 16, 2020
Feb 24, 2021
Jun 21, 2021
Ghana samples tested
Senegal tests performed
Gambia samples tested
Nigeria samples tested
Source: Official data collated by Our World in Data – Last updated 22 June, 11:10 (London time)
OurWorldInData.org/coronavirus • CC BY
Note: Comparisons of testing data across countries are affected by differences in the way the data are reported. Daily data is interpolated for countries not reporting testing data on a daily basis. Details can be found at our Testing Dataset page.
d
Daily COVID-19 tests per thousand people
The figures are given as a rolling 7-day average.
Our World in Data
4
3
2
1
0
Mar 6, 2020
Aug 8, 2020
Nov 16, 2020
Feb 24, 2021
Jun 21, 2021
Switzerland tests performed
Ghana samples tested
Senegal tests performed
Gambia samples tested
Nigeria samples tested
Source: Official data collated by Our World in Data – Last updated 22 June, 11:10 (London time)
OurWorldInData.org/coronavirus • CC BY
Note: Comparisons of testing data across countries are affected by differences in the way the data are reported. Daily data is interpolated for countries not reporting testing data on a daily basis. Details can be found at our Testing Dataset page.
