# Supplementary material for: Detailed phylogenetic analysis tracks transmission of distinct SARS-COV-2 variants from China and Europe to West Africa
Source: Sci Rep. 2021 Oct 26;11:21108. doi: 10.1038/s41598-021-00267-w (PMC8548492; doi:10.1038/s41598-021-00267-w)
Supplement: Supplementary file 6 — Supplementary Table 1. [file 41598_2021_267_MOESM6_ESM.docx]

**Supplementary Table 1: Case fatality and percentage of mutation D614G in West African countries**

| country | deaths | cases | case fatality (%) | D614G (%) |
| --- | --- | --- | --- | --- |
| Senegal | 272 | 13013 | 2.09 | 82.61 |
| Gambia | 87 | 2685 | 3.24 | 66.67 |
| Ghana | 261 | 43505 | 0.60 | 53.33 |
| Nigeria | 1002 | 52227 | 1.92 | 26.32 |
|  |  |  | Pearson correlation  r(% D614G, % case fatality): | 0.28 |

(Retrieved from the Johns Hopkins University corona map https://coronavirus.jhu.edu/map.html, 08/24/20)
